# Supplementary material for: A Behavioural-Theory-Based Qualitative Study of the Beliefs and Perceptions of Marginalised Populations towards Community Volunteering to Increase Measles Immunisation Coverage in Sabah, Malaysia
Source: Vaccines (Basel). 2023 Jun 2;11(6):1056. doi: 10.3390/vaccines11061056 (PMC10301022; doi:10.3390/vaccines11061056)
Supplement: Supplementary file 1 [file vaccines-11-01056-s001.zip › vaccines-2333952-supplementary.pdf]

INTERVIEW GUIDELINES

INTRODUCTION

You are invited to participate in a study titled "A Qualitative Study Using Behavioural Theory to Explore the Marginalised Population's Beliefs and Perceptions Regarding Community Volunteering Methods to Increase Measles Immunisation Coverage in Sabah, Malaysia."

PURPOSE OF THE STUDY

This study was conducted to find out in depth your opinion about the acceptance of community-based measles intervention in the community. In addition, this study also aims to identify your knowledge about measles, risk factors and the probability that you and your family can be exposed to measles. Finally, this study wants to know if you have a new idea to realize a community-based measles intervention in the community in order to benefit more people.

One of the important goals of this research is to share opinions and ideas to carry out community-based measles intervention in the community. The results of this study will be communicated to the parties in charge in order to improve existing weaknesses in increasing measles immunization coverage in the state of Sabah.

RESEARCH PROCEDURE

If you agree to participate in this study, your cooperation is expected to be fully involved in this study.

1. Spend 20-30 minutes participating in a face-to-face session.

2. Give permission to record the audio of the discussion that will be conducted individually.

SIDE EFFECTS AND RISKS

This study will not give you any serious side effects or dangerous risks.

BENEFITS

This survey is important so that we can know your thoughts and ideas about community-based measles interventions. This will be helpful in conducting community-based measles interventions in the future. With this intervention, we will be able to help increase measles immunization coverage for the benefit of communities in need.

PARTICIPATION IN THE STUDY

Your participation in this study is voluntary and there is no coercion. Even if you have agreed to participate in this study and have signed the consent form for this study, you may choose to withdraw or end participation in the study at any time. You do not have to give any reason and there will be no penalty if you end your participation in this study.

QUESTION

If there are any questions or concerns regarding this study, please contact the chief researcher Dr Hazeqa Bte Mohamed Salleh via personal number 011-0235619.

STUDY COST

Your participation in this study is voluntary.

CONFIDENTIAL

All information and results from this study are confidential and confidentiality will be strictly maintained to the extent permitted by law. Your information will not be used for other purposes or disclosed to other parties without permission. The results of this study will be analyzed as a group of data and this data will be given to University Malaysia Sabah (UMS) for research purposes. Additionally, there may be a need for a representative of the Ethics Committee to access your data to verify the information collected for this study. In such circumstances, confidentiality will be maintained at all times. Your data will not be used for other purposes or disclosed to other parties without permission. The results of this research may be published and given to the relevant departments in Malaysia.

TAKING PART IN THE STUDY

If you agree to participate in this study, you will be asked to sign a Consent Form stating that you have been informed about this study, fully understand the explanation from the researcher and agree to participate in this study voluntarily. By signing the Consent Form, your legal rights will not change. Before deciding to participate in this study, you may want to discuss this with another relative or friend. A copy of the consent form will be given to you for reference and storage.

TAJUK KAJIAN / ***RESEARCH TITLE*****:**

A Qualitative Study Using Behavioural Theory to Explore the Marginalised Population's Beliefs and Perceptions Regarding Community Volunteering Methods to Increase Measles Immunisation Coverage in Sabah, Malaysia

Kajian Kualitatif Menggunakan Teori Tingkah Laku untuk Meneroka Kepercayaan dan Persepsi Penduduk Terpinggir Berkenaan Kaedah Sukarelawan Komuniti untuk Meningkatkan Liputan Imunisasi Campak di Sabah, Malaysia.

BAHAGIAN A / PART A

MAKLUMAT PESERTA / PARTICIPANT'S INFORMATION

Nama/*Name* : ____________________________________ Umur/*Age* : _____ Jantina/*Sex* : _________

Pekerjaan/ *Occupation* : _____________________________

Tahap Pendidikan Ibu / *Mother's Education level*

(Sila tandakan pada jawapan yang berkenaan/*Please tick on the related answer*)

Tiada / None		Sekolah menengah / secondary education	
Sekolah rendah / Primary education		Pengajian tinggi / tertiary education	

Immunization history of child and any other children */ Sejarah imunisasi anak-anak* :

(Sila tandakan pada jawapan yang berkenaan/*Please tick on the related answer*)

1st child	( ) Immunized ( ) Not Immunized
2nd child	( ) Immunized ( ) Not Immunized

3rd child	( ) Immunized ( ) Not Immunized
4th child	( ) Immunized ( ) Not Immunized
5th child	( ) Immunized ( ) Not Immunized

BAHAGIAN B / PART B

SOAL SELIDIK / QUESTIONNAIRE

1. Sila huraikan pandangan / pendapat peribadi anda melalui kefahaman anda mengenai demam campak

   Please describe your personal view / opinion within your understanding in regards to measles disease.

   ____

   ____

   ____

   ____

2. Adakah anda dan keluarga terdedah kepada jangkitan demam campak?

   Are you and your family susceptible towards measles disease?

   ____

   ____

   ____

   ____

3. Sila huraikan pandangan / pendapat anda mengenai risiko di mana anda dan keluarga boleh mendapat demam campak?

   What is your opinion regarding the risk of you and your family getting measles disease?

____

____

____

____

4. Sila huraikan pandangan / pendapat anda mengenai faktor yang menhalang anda dari mendapatkan perkhidmatan berasakan komuniti untuk imunisasi demam campak?

   Please describe your personal view / opinion regarding factors that prevent you from getting measles immunisation?

   ____

   ____

   ____

   ____

5. Siapakah yang membuat keputusan tentang kesihatan anak-anak anda?

   Who normally makes decision about your children's health?

   ____

   ____

   ____

   ____

6. Siapakah yang anda rujuk jika tidak pasti apa yang perlu dilakukan mengenai imunisasi anak-anak anda?

   Who do you typically ask if you are not sure what to do regarding your children's immunization?

____

____

____

____

7. Adakah anda serta keluarga menerima perkhidmatan berasaskan komuniti untuk penyakit demam campak?

Do you think that you and your family will accept a measles community-based participation program?

____

____

____

____

8. Pada pandangan anda, bagaimana perkhidmatan berasaskan komuniti dapat membantu anda dan keluarga dalam mendapatkan imunisasi demam campak?

In your opinion, how do you think that community-based participation can help you and your family obtain the measles immunisation?

____

____

____

____

9. Pada pandangan anda, apakah faedah yang anda dan keluarga boleh peroleh dari perkhidmatan berasaskan komuniti untuk imunisasi demam campak?

What is your opinion on the benefits of measles community-based participation program that you and your family can gain?

____

____

____

____

10. Apakah harapan dan cadangan anda untuk perkhidmatan berasaskan komuniti dijalankan di kawasan anda, agar aktiviti ini dapat memberikan manfaat kepada lebih ramai orang?

What are your hopes and ideas for a measles community-based participation program to be done in your area to ensure this activity can benefit more people?

____

____

____

____
